# A quantitative evaluation of aerosol generation during tracheal intubation and extubation

**DOI:** 10.1111/anae.15292

**Published:** 2020-10-22

**Authors:** J. Brown, F. K. A. Gregson, A. Shrimpton, T. M. Cook, B. R. Bzdek, J. P. Reid, A. E. Pickering

**Affiliations:** ^1^ Department of Anaesthesia and Intensive Care Medicine North Bristol NHS Trust Bristol UK; ^2^ School of Chemistry University of Bristol Bristol UK; ^3^ School of Physiology, Pharmacology and Neuroscience University of Bristol Bristol UK; ^4^ Department of Anaesthesia and Intensive Care Medicine Royal United Hospital NHS Trust Bath UK; ^5^ University Hospitals Bristol Bristol UK

**Keywords:** aerosol‐generating procedure, COVID‐19, extubation, intubation, SARS‐COV‐2

## Abstract

The potential aerosolised transmission of severe acute respiratory syndrome coronavirus‐2 is of global concern. Airborne precaution personal protective equipment and preventative measures are universally mandated for medical procedures deemed to be aerosol generating. The implementation of these measures is having a huge impact on healthcare provision. There is currently a lack of quantitative evidence on the number and size of airborne particles produced during aerosol‐generating procedures to inform risk assessments. To address this evidence gap, we conducted real‐time, high‐resolution environmental monitoring in ultraclean ventilation operating theatres during tracheal intubation and extubation sequences. Continuous sampling with an optical particle sizer allowed characterisation of aerosol generation within the zone between the patient and anaesthetist. Aerosol monitoring showed a very low background particle count (0.4 particles.l^−1^) allowing resolution of transient increases in airborne particles associated with airway management. As a positive reference control, we quantitated the aerosol produced in the same setting by a volitional cough (average concentration, 732 (418) particles.l^−1^, n = 38). Tracheal intubation including facemask ventilation produced very low quantities of aerosolised particles (average concentration, 1.4 (1.4) particles.l^−1^, n = 14, p < 0.0001 vs. cough). Tracheal extubation, particularly when the patient coughed, produced a detectable aerosol (21 (18) l^−1^, n = 10) which was 15‐fold greater than intubation (p = 0.0004) but 35‐fold less than a volitional cough (p < 0.0001). The study does not support the designation of elective tracheal intubation as an aerosol‐generating procedure. Extubation generates more detectable aerosol than intubation but falls below the current criterion for designation as a high‐risk aerosol‐generating procedure. These novel findings from real‐time aerosol detection in a routine healthcare setting provide a quantitative methodology for risk assessment that can be extended to other airway management techniques and clinical settings. They also indicate the need for reappraisal of what constitutes an aerosol‐generating procedure and the associated precautions for routine anaesthetic airway management.

## Introduction

The severe acute respiratory syndrome coronavirus‐2 (SARS‐CoV‐2) and associated coronavirus disease 2019 (COVID‐19) pandemic have had an unprecedented impact on global health and the world economy. Drastic interventions to limit transmission have been introduced worldwide, such as lockdowns, physical distancing and the use of personal protective equipment (PPE). Respiratory secretions have a high SARS‐CoV‐2 viral load and are believed to be the main source for person‐to‐person transmission [1, 2, Cevik et al., preprint: doi.org/10.1101/2020.07.25.20162107]. Coughing and sneezing atomise respiratory secretions into particles with different aerodynamic properties according to size; particles greater than 20 µm in diameter are conventionally defined as droplets and tend to follow a ballistic trajectory. These droplets can either directly contact and infect a susceptible individual within close proximity or may settle on nearby surfaces (fomites) where viable virus can exist for up to 72 h [2, 3]. This direct droplet and indirect contact transmission are considered the predominant modes of spread of SARS‐CoV‐2, providing the rationale for physical distancing and hand hygiene as primary measures to reduce the incidence of COVID‐19.

The extent to which SARS‐CoV‐2 is transmitted by the airborne route is a controversial topic [3, 4, 5, 6]. Aerosolised particles (typically considered to be < 20 µm in diameter and particularly those of < 5 µm) may transmit infection by deposition on respiratory epithelium and can potentially transit the full extent of the respiratory tract. It is also feared that these small particles may remain airborne for long periods and may be carried far from the site of origin by air currents. The risks from aerosols and optimum methods of preventing transmission are under active debate [7, 8, 9, 10]. To minimise airborne transmission of SARS‐CoV‐2 to healthcare workers, specific patient care activities have been designated as aerosol‐generating procedures. Tracheal intubation and extubation, manual ventilation via facemask and respiratory tract suctioning are all designated as aerosol‐generating procedures [11, 12, 13]. Many organisations, including the World Health Organization and the public health bodies of the UK, have recommended that those undertaking these aerosol‐generating procedures wear airborne precaution PPE consisting of a fitted face‐piece (FFP3 or NR95), a long sleeved, fluid‐resistant gown, gloves and eye protection [1, 13].

The quantitative evidence base for these guidelines is weak and the relative magnitude of risk for each aerosol‐generating procedure is unknown [3, 11]. The evidence for this designation is largely based on retrospective cohort and case‐controlled studies of transmission during the severe acute respiratory syndrome (SARS) pandemic in 2003 [12, 14]. A systematic review of these studies concluded that tracheal intubation was associated with a significant increase in risk of disease transmission but categorised the quality of available evidence as “*very low quality based on GRADE*” and identified that “*a significant research gap exists in the epidemiology of the risk of transmission of acute respiratory infections from patients undergoing aerosol generating procedures to healthcare workers, and clinical studies should be carefully planned to address specific questions around the risks of transmission in these settings*” [12]. An attempt to provide such evidence employed aerosol sampling traps placed in the vicinity of patients with H1N1 influenza during periods of hospital care, including some with aerosol‐generating procedures, but this large study did not clearly demonstrate an increased risk above background of detecting virus RNA in the air [15]. There is still no quantitative evidence of increased aerosol generation from the designated aerosol‐generating procedures, which likely relates to the challenge of obtaining such measurements in routine healthcare settings.

When considering the risk of transmission of SARS‐CoV‐2, it is helpful to reflect on the definition of an aerosol‐generating procedure that has been expressly stated as “*aerosol generating procedures are considered to have a greater likelihood of producing aerosols compared to coughing*.” [16]. There is a comparatively large quantitative evidence base around aerosolised particle generation by coughing from laboratory‐based investigations with sizes ranging from visible droplets to submicron particles [17, 18, 19, 20]. Given the uncertain balance of potential risks and benefits associated with the protective strategies put in place to limit viral transmission, it is important to quantitatively assess the degree to which individual aerosol‐generating procedures generate aerosolised particles. In this study, we have quantitated airborne particle emission in real‐time during tracheal intubation and extubation, using particle analysis instruments in a working operating theatre environment and compared this with volitional coughs as a reference.

## Methods

A prospective environmental monitoring study was conducted to quantitate the airborne particle size distribution and particle number concentration produced by aerosol‐generating procedures in four operating theatres in a UK hospital (North Bristol NHS Trust). Institutional Review Board approval for the study was given by the Faculty of Life Science and Science Research Ethics Committee at the University of Bristol. As this was an observational study, the anaesthetic and theatre team undertook their normal practice during airway management. The researchers were not involved in the delivery of anaesthetic care.

Observations were made within operating theatres with an ultraclean, laminar flow ventilation system (EXFLOW 32; Howorth Air Technology, Farnworth, UK) with high efficiency particulate air (HEPA) filtration. This ventilation system has a canopy ‘clean zone’ where surgical procedures are performed; the air circulation velocity is 0.2 m.s^−1^ at 1 m above the floor below the canopy and produces 500–650 air changes per hour. All aerosol recordings were performed under the canopy. Air temperature in theatres was set to 20 °C and humidity between 40 and 60%.

A lightweight, portable Optical Particle Sizer (TSI Incorporated, model 3330, Shoreview, NM, USA) was used which samples air at 1 l.min^−1^ and detects particles by laser optical scattering. The optical particle sizer reports the particle number concentration and size distribution within the diameter range 300 nm to 10 µm with a time resolution of 1 s. It is widely used for aerosol studies both within laboratories and clean rooms to use in more demanding applications in the outside environment. It is calibrated by the manufacturer using polystyrene latex spheres and its performance conforms to the ISO standard 21501‐4:2018. A sampling funnel was 3D printed (RAISE3D Pro2 Printer, 3DGBIRE, Chorley, UK) with a maximum diameter of 150 mm, cone height of 90 mm with a 10‐mm exit port. A conductive silicone sampling tube of 2 m length and internal diameter 4.8 mm (3001788, TSI) connected the sampling funnel to the optical particle sizer. This had an internal volume of 145 ml giving a transit lag between the funnel and the particle sizer (with a flow of 1 l.min^−1^) of 8.7 s which was taken into account in the time registration of measurements.

**Figure 1:**
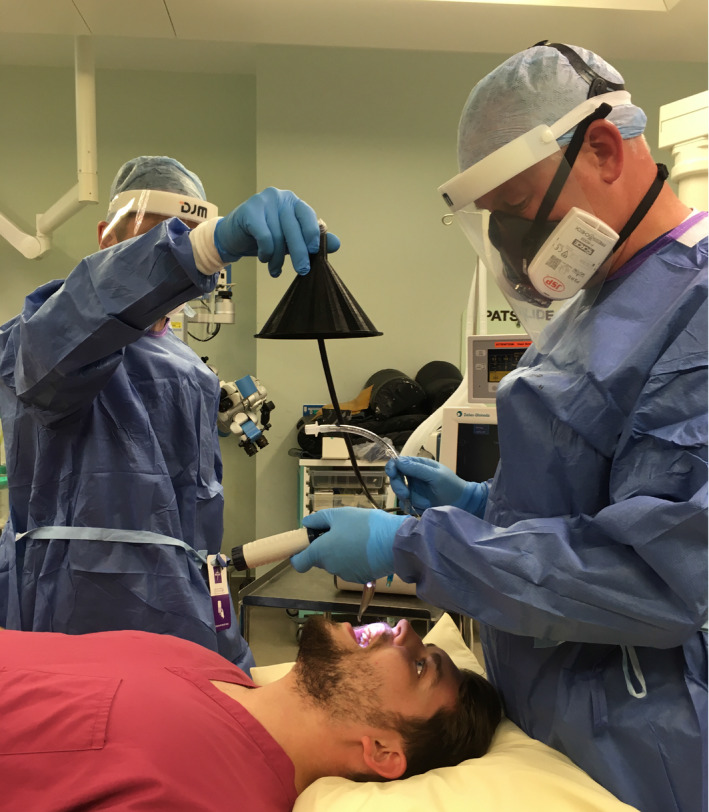
Simulation of aerosol measurement approach within operating theatre environment. The sampling funnel was positioned 0.5 m above the source of aerosol in the airway management zone allowing a sampling stream of air (1 l.min^−1^) to be routed to the optical particle sizer.

In an initial set of pilot studies in the ultraclean theatre environment, it was possible to reliably detect a volitional cough from a subject lying supine (JB) at a sampling distance of 0.5 m from the mouth to the funnel. Sampling at 0.5 m approximates the distance from the face of the airway management practitioner to the patient's mouth during the intubation sequence. For recordings during intubation and extubation, the sampling funnel was, therefore, positioned at a distance of 0.5 m facing the patient's mouth and close to the anaesthetist (Fig. 1). For several extubations, we repositioned the funnel to lie above and behind the patient's head, again at 0.5 m and facing the patient's airway. The funnel was handheld to ensure it could be quickly removed from the airway management zone in case of clinical need (this was not needed in the course of the study). All healthcare workers, and members of the investigating team, wore airborne precaution PPE during aerosol‐generating procedure measurements.

Tracheal intubation and extubation consist of a series of discrete events and procedures which we designated as sequences. For tracheal intubation measurements, anaesthetic induction followed a conventional sequence with pre‐oxygenation, intravenous induction by administration of anaesthetic and neuromuscular blocking drugs, manual ventilation of the lungs via a facemask, direct laryngoscopy and intubation of the trachea followed by inflation of the tracheal tube cuff which was the reference end point of the intubation sequence. Standard anaesthetic monitoring was used including waveform capnography. This whole intubation sequence typically lasted 3–4 min with continuous aerosol monitoring throughout. The 5‐min period before inflation of the tracheal tube cuff was analysed.

For recordings during tracheal extubation, the level of anaesthesia was lightened, spontaneous breathing allowed to recommence, the oropharynx was suctioned before the tracheal tube cuff was deflated, and the trachea was then extubated according to the anaesthetist's normal practice. After extubation and confirmation of airway patency, the patient received oxygen via an anaesthetic facemask and then a Hudson™ mask. The reference point for the start of extubation was tracheal tube cuff deflation (releasing the seal on the airway). Continuous monitoring with the optical particle sizer was conducted throughout and a period of 3 min before and up to 2 min after cuff deflation was analysed.

Airway management events including cuff inflation, deflation and coughs were recorded contemporaneously by the researcher using a time stamp application (Emerald Timestamp, Emerald Sequoia LLC, https://emeraldsequoia.com/ts/index.html). Data were exported from the TSI optical particle sizer, processed in the TSI Aerosol Instrument Manager software, and analysed in Origin Pro (Originlab, Northampton, MA, USA) and Prism v8 (Graphpad, San Diego, CA, USA). Comparisons were made between aerosol‐generating events with unpaired t‐tests with the significance level set at p < 0.05.

## Results

**Figure 2:**
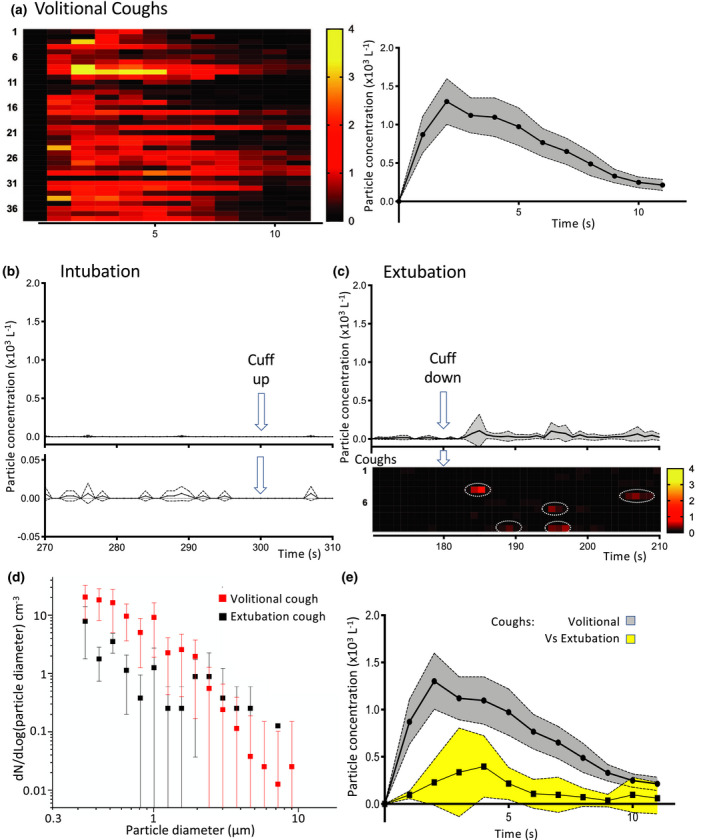
Aerosol measurements during intubation and extubation in operating theatre environment. (a) Temporal profile of aerosol generation from volitional coughs. Individual recordings (n = 38) represented on heat map showing the total number particle concentration over time. Average time course plotted (mean with 95%CI) showing a peak after 2 s and a rapid decay back to baseline. (b) Profile of the total number concentration of aerosol detected during the critical phase of intubation (arrow at 300 s marks completion of intubation with cuff up). When plotted on the same scale as the cough (b) then this looks essentially flat and when shown on a ten‐fold expanded scale below it can be seen that it is not significantly different to baseline as the confidence intervals always span zero (mean ± 95%CI). (c) Extubation recordings from each patient (n = 10) plotted as the average and individually as rows on heat map of number concentration of particles (lower, on same scale as b). This showed sporadic aerosol events (red, ringed) after cuff deflation set on a low baseline level of particles. The average concentration of aerosol shown above was low overall (mean ± 95%CI). (d) The extubation cough events (n = 5) had a similar aerosol particle size distribution to volitional coughs with a predominance of diameters < 1 µm (mean ± SD). (e) The extubation coughs were of a smaller magnitude than the volitional coughs (particle number concentration profile shown overlaid, mean ± 95%CI).

Environmental aerosol monitoring was conducted over a 3‐week period during operating lists for orthopaedic trauma and neurosurgical emergencies. Recordings were made of 19 intubations and 14 extubations. The conduct of anaesthesia was left at the discretion of the anaesthetist, who ranged in experience from junior trainee to senior consultant. Control environmental monitoring recordings showed the ultraclean, laminar flow ventilation and air filtration system produced a very low background of aerosol particles averaging 0.4 l^−1^ when the theatre was empty, and 3.4 l^−1^ when the theatre was in use but no aerosol‐generating procedures were in progress. Thirty‐eight volitional coughs were sampled at 0.5‐m distance. These coughs showed a characteristic profile with a rapid and transient spike of expectorated particles (Fig. 2a). Peak aerosol concentration occurred 2 s after the cough was registered and averaged 1310 (905) particles.l^−1^. The spike in aerosol particle count decayed back to baseline with a time constant of approximately 2.7 s. Each cough contained an average of 134 (77) detected airborne particles (over the 12 s window). The large majority of the particles were < 1 µm diameter (Fig. 2d). Although we conducted our monitoring under the ultraclean ventilation canopy, the temporary suspension of the laminar flow system (0.2 m.s^−1^) did not alter the number of particles detected per cough: 164 (80) with ventilation on vs. 153 (82) with ventilation off (p = 0.77, n = 9 per group).

All tracheal intubation sequences included manual facemask ventilation and three also required repeated attempts at laryngoscopy/intubation. The mean (SD) number of particles detected in a 5‐min period during anaesthetic induction and intubation was 7 (6) (n = 14), compared with a background in the empty theatre of approximately two particles per 5‐min period (Fig. 2b). The average concentration of particles recorded during the intubation period (1.4 (1.4) l^−1^, n = 14) was 500‐fold lower than the mean (SD) concentration recorded during volitional coughs (732 (418) particles.l^−1^, n = 38, p < 0.0001). Similarly, the maximum concentration recorded during intubation averaged across events (77 (49) particles.l^−1^, n = 14) was 22‐fold lower than that seen with volitional coughs (1688 (872), n = 38, p < 0.0001). An equivalent series of aerosol measurements during tracheal intubation with laminar flow ventilation suspended produced a similar particle count to that seen in the presence of flow (6 (2) particles with flow off, n = 5, p = 0.65 vs. flow on).

Extubation produced a mean (SD) concentration of aerosolised particles of 21 (18) l^−1^ (n = 10, Fig. 2c) which was 35‐fold lower than that seen during a volitional cough (p < 0.0001) but 15‐fold greater than that seen during intubation (p = 0.0004). The maximum concentration recorded during extubation averaged across events (432 (209) particles.l^−1^, n = 10) was lower than that seen with volitional coughs (1688 (872), n = 38, p < 0.0001). The average total number of particles detected during the period of extubation was 100 (85). This is similar to that detected during a single volitional cough, although sampling during extubation summed particles produced over 5 min and each cough over 12 s. During four of the ten extubations, the patient coughed at least once (typically after tube removal) (Fig. 2c). These extubation coughs produced aerosolised particles of a similar size distribution to the reference coughs (Fig. 2d) but were always smaller in magnitude than the average volitional cough and, on average, produced only a quarter of the number of particles: 33 (10) (from a total of five coughs during four extubations, Fig. 2e). Because the usual position for the anaesthetist during extubation is to stand above and behind the patient, who is typically semi‐recumbent, a further set of aerosol recordings were made with the particle collector located in that position (0.5 m away and still facing towards the patient). Aerosol monitoring from that position during extubation (three with cough events) greatly decreased the concentration of detected airborne particles to close to background levels seen in an active theatre (3.7 (5.9) l^−1^).

## Discussion

We conducted aerosol sampling in an ultraclean operating theatre environment during routine clinical practice enabling quantitative measurement of aerosols produced by tracheal intubation and extubation. Using the quantity and concentration of aerosolised particles generated by volitional coughs as a reference, we have shown that both intubation and extubation sequences produce less aerosol than voluntary coughing. For the sequence of tracheal intubation, in particular, the concentration of aerosol generated is several orders of magnitude less than a single cough and is only very modestly above background levels of circulating particles in an ultraclean theatre. These findings demonstrate that the process of tracheal intubation is associated with a very low risk of aerosol generation.

Standard anaesthetic induction and intubation sequences are designed to obtund airway reflexes and the use of a neuromuscular blocking drug ensures that the anaesthetised patient can neither breathe nor cough. Of note, we detected no increases in aerosolised particles above the patient's face during anaesthesia, facemask ventilation, airway suction and, on occasion, several repeated attempts at intubation. This reflects typical clinical practice by anaesthetists with a range of experience, providing further reassurance regarding the low level of aerosol generation.

A more nuanced picture is seen during the tracheal extubation sequence where aerosol concentration was greater than that seen with intubation but substantially less than a single cough. The total number of expectorated airborne particles (over a 5‐min period) was similar to a single volitional cough. Indeed, a cough event was noted clinically in 50% of extubations and this was frequently detected as an aerosol spike. These extubation coughs produced a similar particle size distribution but there were fewer airborne particles than with volitional coughs (approximately 25%). Extubation cough aerosol was also transient and only detectable for approximately 5 s. Therefore, it would appear that aerosol generation during extubation with a cough is quantitatively different from extubation without a cough. Although a cough at extubation may be interpreted as a positive sign signalling the return of protective airway reflexes, it is likely to increase the risk of aerosolised particle generation. Therefore, mitigation strategies should be considered to reduce the risk of coughing and exposure to aerosols. As sampling showed much reduced particle numbers behind the patient's head compared with above their airway, clinician exposure could be reduced by the simple expedient of standing behind the patient's head (as is conventional) and, thus, out of the direct stream of any potential cough plume. Coughing on extubation could also be minimised by modifying the anaesthetic technique in higher risk patients [21].

The combination of monitoring within an ultraclean ventilation theatre and the use of a highly sensitive optical particle sampler has afforded sufficient resolution to quantitate aerosol generation in real time during anaesthetic delivery. To put this in context, it is worth noting that we are unaware of any previous recording of aerosol generated even by coughing in a routine healthcare environment as this normally requires highly specialised and controlled laboratory conditions [17, 18, 19, 20]. The ultraclean laminar flow ventilation system theatre had a very low level of airborne particles (0.4 l^−1^); in comparison the baseline aerosolised particle concentration in a nearby non‐laminar theatre was more than 3 orders of magnitude higher (15 × 10^3^ l^−1^), which would have precluded detection of aerosols generated either by tracheal intubation or extubation (and perhaps even a cough). To assess the impact of the laminar flow on our observations, we undertook measurements with the ultraclean theatre ventilation on and off both for coughing and for tracheal intubation and these did not differ. This demonstrates that the low aerosol particle counts were not secondary to immediate aerosol clearance by high laminar ventilation flows. Although we did not assess the impact of ventilation flow on the aerosol generated during extubation (because of the pragmatic issue of turning off the ventilation while the case is in theatre and the perceived need to disperse any accumulated aerosol) we have no reason to believe, based on the cough and intubation measurements, that the presence of laminar flow has materially influenced these extubation measurements.

There are a number of limitations to the study and these include a relatively small number of observations, the use of pragmatic design without control over the specific anaesthetic administered or the grade of practitioner and sampling aerosol from a limited arc encompassed by the funnel. The reference coughs were from a single subject (one of the investigators), but data from another study (Brown et al., unpublished data) indicate that they were not outliers when compared with other healthy subjects. Additionally, we have made no measurements from subjects known to have COVID‐19 or other intercurrent respiratory comorbidity, which would be an important area for investigation, although challenging to conduct. The measurements were taken during anaesthesia for patients receiving urgent orthopaedic and neurosurgical interventions and may not be generalisable to intubations in a critical care/emergency setting that may be conducted in extremis.

Importantly, it should be acknowledged that we are unable to make any conclusion about the risk of actual SARS‐CoV‐2 transmission as aerosol generation is still only a presumed risk‐factor and particle number concentration is a plausible but unproven surrogate measure of that infection risk. We have presented our data as mean particle concentration during the event, maximum concentration recorded during the epoch and total numbers of detected airborne particles and note that there is no available evidence to indicate which measure will prove to be the best surrogate measure of infection risk [3]. Other dimensions that are likely to be important are the size distribution of the aerosolised particles (which influences their airborne transport and ability to be carried into the respiratory tract), the total volume of expectorate (which can be derived if assumptions about sphericity and composition are made) and the concentration of live virions within the particles (not measured here). Each measure has limitations, but we consider that the average concentration over time gives the best estimate of the relative exposure smoothed over the at‐risk period during which particles may be inhaled or deposited on mucous membranes. We note that the peak concentration is likely to overestimate the risk; for example, a single particle detected in a 1‐s time bin during an intubation sequence (5‐min period of recording) would correspond to a maximum of 60 particles.l^−1^ (with 1 l.min^−1^ air flow through the particle detector). However, this is probably better represented as the average concentration of 0.2 particles.l^−1^ when assessing the relative risk over time, reflecting the fact that no particles are detected for 299 of the 300 s recording period. Notwithstanding these considerations, we believe that our aerosol measurements constitute a valuable quantitative dataset and we note that the methodology could be applied to other anaesthetic airway management techniques and designated aerosol‐generating procedures to extend the relative risk ranking.

Our results for the risk of aerosol generation associated with tracheal intubation are at odds with previous retrospective evidence that was used to designate intubation in an aerosol‐generating procedure [12, 14]. These studies found an association between acquiring SARS and being in the room during intubation but without any measure of aerosol generation. It is difficult to directly compare these two sources of evidence: in our study, all patients received a controlled anaesthetic induction that included a neuromuscular blocking drug. Conversely, during the SARS epidemic patients were unwell, may have been coughing during the intubation sequence and it is likely that viral secretion was at peak levels at the point of initiating intensive care management (which is not the case for COVID‐19 Cevik et al., preprint: doi.org/10.1101/2020.07.25.20162107). It is equally plausible that other mechanisms of transmission, such as direct exposure to respiratory secretions or fomites or association between those who undertook tracheal intubation and performance of other high‐risk activities, could have contributed to the spread of SARS.

By the definition noted earlier, aerosol‐generating procedures are considered to have a greater likelihood of producing aerosols compared with coughing [16]. Our study indicates that the process of elective tracheal intubation produces a barely recordable increase in aerosol and, consequently, should not be designated as an aerosol‐generating procedure. When a patient coughs during tracheal extubation, a measurable particle plume is produced but the aerosol is still smaller than a single volitional cough. These relative risks aee4 of aerosol generation need to be balanced against the knowledge that the use of airborne precaution PPE has substantial impact on clinical practice. Additionally, methods introduced to mitigate the risks posed by bio‐aerosols have reduced operating theatre turnover, decreased hospital productivity and increased waiting times for elective and cancer surgery. A further important consideration relates to the cost and limited supply of PPE which has to be targeted to appropriate healthcare settings on the basis of risk. These results, therefore, should help inform future airborne prevention PPE guidelines by providing evidence on the relative risk of aerosol generation associated with tracheal intubation and extubation.

## References

[1] Cook TM . Personal protective equipment during the coronavirus disease (COVID) 2019 pandemic – a narrative review. Anaesthesia 2020; 75: 920–7. 32246849 10.1111/anae.15071

[2] van Doremalen N , Bushmaker T , Morris DH , et al. Aerosol and surface stability of SARS‐CoV‐2 as compared with SARS‐CoV‐1. New England Journal of Medicine 2020; 382: 1564–7. 32182409 10.1056/NEJMc2004973 PMC7121658

[3] Wilson NM , Norton A , Young FP , Collins DW . Airborne transmission of severe acute respiratory syndrome coronavirus‐2 to healthcare workers: a narrative review. Anaesthesia 2020; 75: 1086–95. 32311771 10.1111/anae.15093 PMC7264768

[4] Miller SL , Nazaroff WW , Jimenez JL , et al. Transmission of SARS‐CoV‐2 by inhalation of respiratory aerosol in the Skagit Valley Chorale superspreading event. Indoor Air 2020. Epub 26 September. 10.1111/ina.12751 PMC7537089 32979298

[5] Morawska L , Milton DK . It is time to address airborne transmission of COVID‐19. Clinical Infectious Diseases 2020. Epub 7 July. 10.1093/cid/ciaa939. PMC7454469 32628269

[6] Schutzer‐Weissmann J , Magee DJ , Farquhar‐Smith P . Severe acute respiratory syndrome coronavirus 2 infection risk during elective peri‐operative care: a narrative review. Anaesthesia 2020. Epub 11 July. 10.1111/anae.15221. PMC7404908 32652529

[7] Asadi S , Bouvier N , Wexler AS , Ristenpart WD . The coronavirus pandemic and aerosols: does COVID‐19 transmit via expiratory particles? Aerosol Science and Technology 2020. Epub 3 April. 10.1080/02786826.2020.1749229 PMC7157964 32308568

[8] Jayaweera M , Perera H , Gunawardana B , Manatunge J . Transmission of COVID‐19 virus by droplets and aerosols: a critical review on the unresolved dichotomy. Environmental Research 2020. Epub 13 June. 10.1016/j.envres.2020.109819. PMC7293495 32569870

[9] Liu Y , Ning Z , Chen Y , et al. Aerodynamic analysis of SARS‐CoV‐2 in two Wuhan hospitals. Nature 2020; 582: 557–60. 32340022 10.1038/s41586-020-2271-3

[10] Zhang R , Li Y , Zhang AL , Wang Y , Molina MJ . Identifying airborne transmission as the dominant route for the spread of COVID‐19. Proceedings of the National Academy of Sciences of the United States of America 2020; 117: 14857–63. 32527856 10.1073/pnas.2009637117 PMC7334447

[11] NHS National Services Scotland and Health Protection Scotland . Aerosol Generating Procedures (AGPs). 2020. http://www.nipcm.hps.scot.nhs.uk/web-resources-container/transmission-based-precautions-literature-review-aerosol-generating-procedures (accessed 08/09/2020).

[12] Tran K , Cimon K , Severn M , Pessoa‐Silva CL , Conly J . Aerosol generating procedures and risk of transmission of acute respiratory infections to healthcare workers: a systematic review. PLoS One 2012; 7: e35797. 22563403 10.1371/journal.pone.0035797 PMC3338532

[13] Public Health England . COVID‐19: personal protective equipment use for aerosol generating procedures. 2020. https://www.gov.uk/government/publications/covid-19-personal-protective-equipment-use-for-aerosol-generating-procedures (accessed 08/09/2020).

[14] Davies A , Thomson G , Walker J , Bennett A . A review of the risks and disease transmission associated with aerosol generating medical procedures. Journal of Infection Prevention 2009; 10: 122–6.

[15] Thompson KA , Pappachan JV , Bennett AM , et al. Influenza aerosols in UK hospitals during the H1N1 (2009) pandemic – the risk of aerosol generation during medical procedures. PLoS One 2013; 8: e56278. 23418548 10.1371/journal.pone.0056278 PMC3571988

[16] Public Health England . Infection control precautions to minimise transmission of acute respiratory tract infections in healthcare settings. 2016. https://www.gov.uk/government/publications/respiratory-tract-infections-infection-control (accessed 08/09/2020).

[17] Stelzer‐Braid S , Oliver BG , Blazey AJ , et al. Exhalation of respiratory viruses by breathing, coughing, and talking. Journal of Medical Virolology 2009; 81: 1674–9. 10.1002/jmv.21556 19626609

[18] Yang S , Lee GW , Chen CM , Wu CC , Yu KP . The size and concentration of droplets generated by coughing in human subjects. Journal of Aerosol Medicine 2007; 20: 484–94. 18158720 10.1089/jam.2007.0610

[19] Johnson GR , Morawska L , Ristovski ZD , et al. Modality of human expired aerosol size distributions. Journal of Aerosol Science 2011; 42: 839–51.

[20] Zayas G , Chiang MC , Wong E , et al. Cough aerosol in healthy participants: fundamental knowledge to optimize droplet‐spread infectious respiratory disease management. BMC Pulmonary Medicine 2012; 12: 11. 22436202 10.1186/1471-2466-12-11 PMC3331822

[21] Tung A , Fergusson NA , Ng N , Hu V , Dormuth C , Griesdale DEG . Medications to reduce emergence coughing after general anaesthesia with tracheal intubation: a systematic review and network meta‐analysis. British Journal of Anaesthesia 2020; 124: 480–495. 10.1016/j.bja.2019.12.041. 32098647

